# A passive and objective measure of recognition memory in mild cognitive impairment using Fastball memory assessment

**DOI:** 10.1093/braincomms/fcaf279

**Published:** 2025-09-01

**Authors:** George Stothart, Sophie Alderman, Oliver Hermann, Sam Creavin, Elizabeth J Coulthard

**Affiliations:** Department of Psychology, University of Bath, Bath BA2 7AY, England, UK; Department of Psychology, University of Bath, Bath BA2 7AY, England, UK; Department of Psychology, University of Bath, Bath BA2 7AY, England, UK; Bristol Medical School, University of Bristol, Bristol BS8 1QU, England, UK; Bristol Medical School, University of Bristol, Bristol BS8 1QU, England, UK

**Keywords:** EEG, dementia, memory, MCI, fastball

## Abstract

As viable pharmacotherapies and blood biomarkers emerge for dementia treatment and screening, there remains a great need for accurate, sensitive biomarkers of cognitive function. We have previously demonstrated that Fastball, a new Electroencephalography (EEG) method for the passive and objective measurement of recognition memory that requires no behavioural memory response or task comprehension, is sensitive to cognitive dysfunction in Alzheimer's disease. Here we present new evidence that Fastball is sensitive to amnestic dysfunction in an earlier stage of the dementia lifecourse, Mild Cognitive Impairment (MCI). 53 MCI patients and 54 healthy older adult (HOA) controls completed a 3-min Fastball task in which they passively viewed rapidly presented images while EEG captured their automatic ability to differentiate between images based on previous exposure. They also completed neuropsychological assessments of memory (Delayed Match to Sample-48), sustained attention (Psychomotor Vigilance Task), and general cognitive function (Addenbrookes Cognitive Exam-iii). Participants were re-tested after 1 year to establish the test-retest reliability of Fastball in HOAs, and the sensitivity of Fastball to cognitive decline in MCI patients, over a 1 year period. Amnestic MCI patients showed significantly reduced Fastball responses compared with non-amnestic MCI patients (*P* = 0.001, Cohen's *d* = 0.98) and HOA controls (*P* = 0.005, Cohen's *d* = 0.64). Regression analyses showed that Fastball EEG responses were selectively predictive of neuropsychological measures of recognition memory and not attention. Between baseline and year one follow-up Fastball showed moderate to good test-retest reliability in HOA controls, and the six MCI-dementia converters showed a trend for lower Fastball responses at baseline which will be confirmed with further longitudinal assessment. Fastball is further validated as a viable method for testing recognition memory in cognitively impaired populations. We have demonstrated that it is selectively predictive of memory dysfunction and not attention or other cognitive functions. It is passive, non-invasive, quick to administer and uses cheap, scalable EEG technology. Fastball is a viable functional biomarker that can help to advance cognitive assessment in MCI.

## Teaser

Stothart *et al*. present evidence that Fastball, a new EEG method for the passive and objective measurement of recognition memory, is sensitive to amnestic dysfunction in Mild Cognitive Impairment and predicts neuropsychological test performance. Fastball is a passive, non-invasive, quick-to-administer, functional biomarker that uses cheap, scalable EEG technology.

## Graphical Abstract

**Graphical Abstract:**
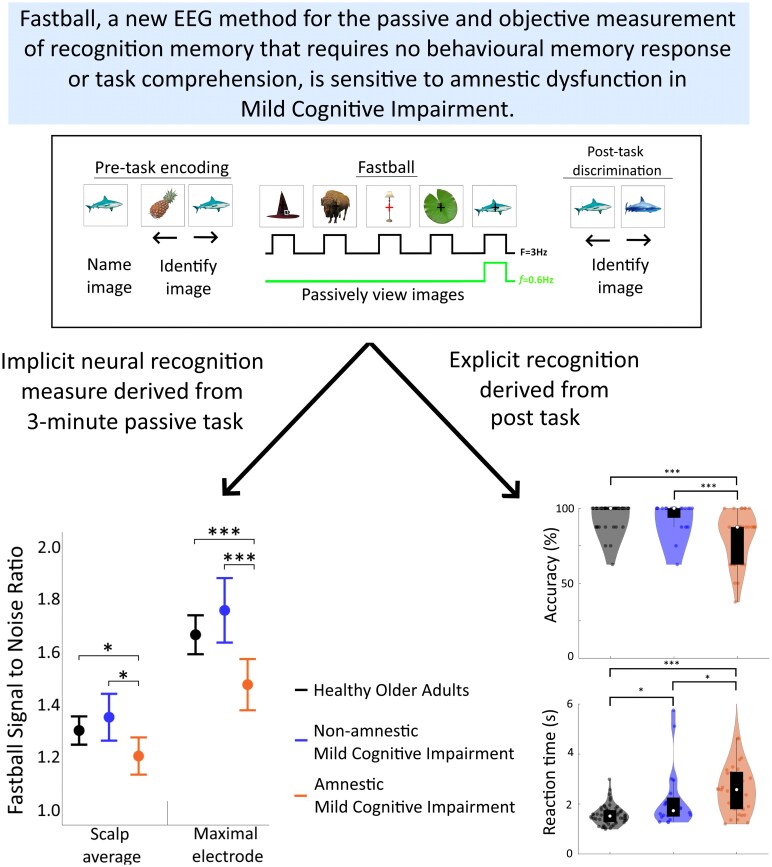

## Introduction

### The need for early diagnosis

Alzheimer's disease (AD) accounts for the majority of dementia cases with a global prevalence estimated at 32 million.^1^ Mild Cognitive Impairment (MCI) is a functionally defined interim stage between normal cognitive function and dementia, with AD is estimated to be the underlying aetiology in 40–75% of cases.^2-4^ Early and accurate diagnostic tools are needed to ensure patients can access treatments earlier and prepare for their future, and the detection of AD amongst MCI patients is a clear and obvious opportunity. New disease-modifying dementia drugs are now available and are most effective when administered at an early stage, making early diagnosis even more vital than before.^5^

MCI patients can be coarsely grouped based on their cognitive impairment into amnestic MCI (aMCI) and non-amnestic MCI (naMCI). In aMCI memory loss is the prominent impairment whereas naMCI patients’ have normal memory function alongside deficits in other cognitive domains such as attention and visuospatial processing.^6^ Longitudinal studies have shown that clinical samples of aMCI progress to AD at a substantially higher rate than naMCI,^7^ with progression rates of around 10–17% per year, 38% after 2.5 years and 40–60% after 4 years.^8-10^

### Current diagnostic tools

AD has historically been diagnosed using a combination of subjective and objective reports of cognitive decline using standardized neuropsychological assessments.^11,12^ More recently a solely biological definition of AD has been recommended for diagnosis with cognitive function used to aid clinical staging,^13,14^ while an alternative definition that includes clinical function as a core feature of the disease has also been proposed.^15^ Positron Emission Tomography (PET) and cerebrospinal fluid biomarkers of amyloid and tau pathology have been the structural biomarkers of choice; however, their use has been hampered by lack of availability, high costs and their invasive nature.^16,17^ Additionally, their relationship with cognition is unclear, as amyloid pathology can present without cognitive decline,^18-20^ and cognitive decline can be present without clinically significant amyloid pathology.^21^ Blood-based biomarkers (BBBMs) may enable earlier detection of amyloid and tau pathology which would allow for earlier diagnosis and help to inform treatment management strategies. The clinical adoption of BBBMs should provide affordable, scalable biomarkers of brain structure with the potential to transform dementia diagnosis, what is lacking is equivalent progress in the assessment of brain function.

Symptoms of cognitive impairment present relatively late in disease progression despite the abnormal accumulation of amyloid and tau pathology occurring in the brain around 10–20 years before the onset of symptoms.^2,3,22,23^ Neuropsychological assessments such as the Addenbrookes Cognitive Examination (ACE)^24^ and Montreal Cognitive Assessment^25^ are used for clinical staging once symptoms are present, but are insensitive to the earliest stages of the disease process. They are also prone to educational and cultural biases,^26,27^ assessment anxiety^28,29^ and require verbal and written communication skills, making them ineffective in populations with language impairments.^30,31^ A more sensitive and equitable test of cognitive function may aid the early detection of AD and allow for targeted treatment. To improve diagnostic accuracy and sensitivity, and aid clinical staging, structural biomarkers may benefit from being combined with early, simple to implement, objective measures of cognitive function that specifically measure AD-related changes.

### Objective measures of cognitive function

Electroencephalography (EEG) can provide a direct measure of neural activity during cognitive tasks. Differences in event-related potentials (ERPs) have been found when comparing AD patients and age-matched control participants, with a reduction and slowing of the P300 response being the most frequently reported outcome.^32,33^ In spite of many decades of positive experimental findings in dementia populations, progression from research to feasible clinical diagnostic tools is yet to be achieved. Barriers include long recording times, which are required to generate data with sufficient signal-to-noise to reliably measure ERPs, and high interindividual variability, which makes it difficult to reliably capture single subject performance. The reliability and reproducibility of ERP results are further impeded by interexperimental variability in how ERP responses are quantified.^34^

### Fastball

Fast periodic visual stimulation (FPVS) is an EEG technique that addresses many of the aforementioned issues. It can be used to objectively measure a range of cognitive functions, has a high signal-to-noise ratio (SNR) and has a short recording time. Importantly, this method does not require task comprehension or a behavioural response, making it a viable tool for assessing cognitive function in patient populations.^35-39^

We recently demonstrated the successful adaptation of FPVS, which we referred to as ‘Fastball’, for the passive and objective measure of recognition memory in AD Patients.^40^ Fastball EEG was sensitive to recognition memory deficits in AD patients compared with healthy older and younger adult controls, with patients being discriminated with high accuracy from controls. Here, we present the application of Fastball in the measurement of recognition memory in MCI.

### Fastball and MCI

Neuroimaging evidence suggests explicit memory (familiarity—the feeling of previously encountering a stimulus, and recollection—explicitly recalling information about a stimulus) is reliant on brain regions known to be affected in early AD, including the perirhinal cortex and hippocampus.^41-45^ Earlier research suggested a clear distinction between implicit (unconscious) and explicit memory, but more recent evidence suggests that they share common memory representations and involve similar medial temporal lobe regions.^46-50^

Healthy older adults (HOAs) generally show impaired recollection and maintained familiarity. AD and MCI patients also show consistent deficits in recollection, with more variation in familiarity performance.^51-53^ Difficulties arise with behavioural tests that measure familiarity in impaired populations due to the subjective interpretation of familiarity requiring a high level of linguistic and semantic function, as well as, biases created through the complexity and comprehension of test instructions.^45^ Recent studies that have delineated the processes of recollection and familiarity judgements have demonstrated more consistent familiarity deficits in aMCI^54,55^ and a longitudinal study demonstrated that familiarity was selectively impaired in aMCI patients that later converted to AD.^56^ Fastball provides a new method of assessing recognition memory that avoids many of the issues with behavioural measures.

### Aims and hypotheses

The current study aims to passively and objectively measure recognition memory in MCI patients using Fastball. In order to further explore the relationship between sustained attention and memory in Fastball, we have included standardized neuropsychological measures of recognition memory and sustained attention.

We predicted aMCI patients would show reduced Fastball responses when compared with HOA and naMCI patients. We predicted that Fastball responses would predict performance on neuropsychological measures of recognition memory, but not sustained attention. We also explored the test-retest reliability of Fastball in HOA, and the sensitivity of Fastball to cognitive decline in MCI patients, over a 1 year period.

## Methods

### Participants

Participant demographics are displayed in Table 1. Fifty-three patients with MCI were recruited on a consecutive incident patient basis from memory clinics in the Southwest of England. The diagnosis of MCI was determined according to NINCDS-ADRDA^12^ guidelines using during expert consensus meetings involving senior neurologists and neuropsychologists using neurological, neuroimaging, physical and biochemical examination together with the results of family interview, neuropsychological and daily living skills assessment.

**Table 1:** Values other than counts indicate means (SD) for baseline and retest participants.

	Healthy older adults (HOA)	aMCI	naMCI	Group comparisons		
				HOA versus aMCI	HOA versus naMCI	aMCI versus naMCI
**n**	54	33	20	-	-	-
**Age**	73(5)	77(8)	73(9)	ns	ns	ns
**Number of females**	31	17	4	ns	0.016	ns
**Years of education**	16(3)	12(2)	14(3)	<0.001	0.045	ns
**ACE-III**						
**Total (/100)**	96(3)	76(7)	90(4)	<0.001	<0.001	<0.001
**Attention (/18)**	17(1)	15(2)	17(1)	<0.001	ns	<0.001
**Memory (/26)**	25(2)	14(4)	24(1)	<0.001	ns	<0.001
**Verbal Fluency (/14)**	13(1)	9(2)	11(2)	<0.001	<0.001	<0.001
**Language (/26)**	26(1)	24(2)	25(2)	<0.001	ns	ns
**Visuospatial (/16)**	16(1)	14(2)	15(1)	<0.001	ns	ns
**Visual acuity (LogMar)** ^a^	0.07(0.13)	0.10(0.12)	0.08(0.12)	ns	ns	ns
**Contrast sensitivity (LogCs)** ^b^	1.85(0.23)	1.70(0.23)	1.76(0.22)	0.008	ns	Ns

HOA and MCI patients were compared using one-way ANOVA with Bonferroni corrected *post hoc* comparisons and sex was compared using a Mann-Whitney U-test. *P* values are indicated in the group comparisons, ns = no significant difference, *P* > 0.05.

ACE-III = Addenbrooke's Cognitive Examination-III.

^a^Higher score = worse acuity.

^b^Higher score = better sensitivity.

MCI patients were split into aMCI and naMCI groups based on a cutoff score of 21 or less on their baseline ACE-iii memory subscale. This cutoff score was two standard deviations below the HOA mean in the current study, see Table 1, and similar to previous studies approaches to defining cognitive impairment relative to age-adjusted norms.^57,58^

Fifty-four HOA were recruited from research volunteer databases maintained by the memory clinics. They were in good general health, had no subjective experience or objective evidence of cognitive impairment or other neuropsychological disorder. Participants were tested at home, using portable EEG systems.

All participants were invited to repeat the study procedure 1 year after the baseline appointment (mean number of days between baseline and re-test at 384 ± 25). Seven aMCI and four naMCI patients did not complete the follow-up assessment due to ill health, loss of capacity to consent, lack of response to the re-test invite and death.

Participants were excluded from the study if they were in poor general health, had a history of epilepsy or seizures, had any other psychiatric or neurological disorder, or were visually impaired. No relationship was found between visual acuity, contrast sensitivity, and the dependent variables in this study.

The research was approved by NHS REC (Ref: IRAS 291799). All participants had capacity to consent and provided written informed consent in accordance with the Declaration of Helsinki. Participants were free to withdraw at any time.

### Materials

#### Fastball stimuli

**Figure 1:**
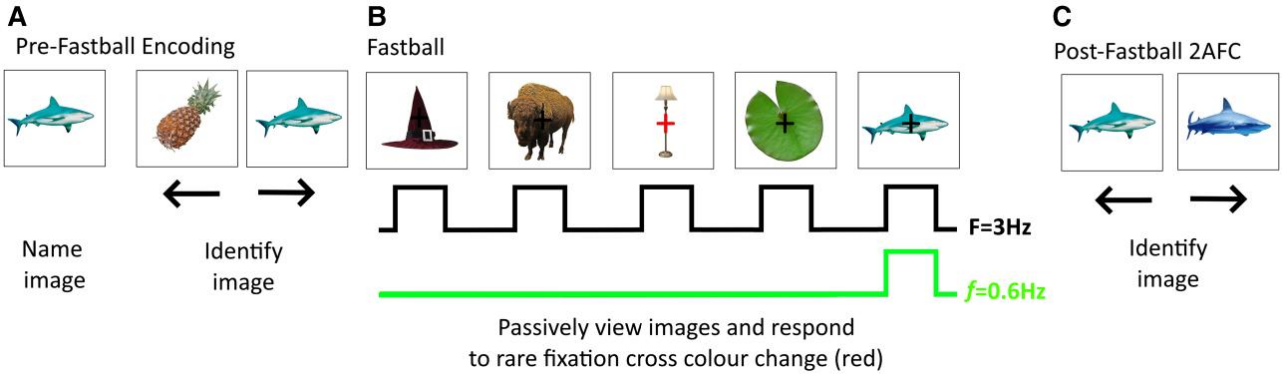
**Fastball task design.** (**A**) Pre-Fastball encoding: Participants were presented with a single image which they named out loud. They then identified it in a 2AFC task, in which it was paired with a previously unseen image (foil). (**B**) Fastball: A visual steady state response (**F**) is elicited in response to the presentation of stimuli at 3 Hz. Black and blue lines indicate the predicted neural response to standard and oddball stimuli. An oddball response *f* is elicited if the oddball stimulus is recognized. Participants attended to a black, central fixation cross and pressed a key in response to it turning red in 10% of standard image sequences. (**C**) Post-Fastball 2AFC: Participants were required to identify the previously seen oddballs and eight randomly pre-selected standard images when displayed next to a similar lure.

Images were chosen from the Bank of Standardized Stimuli v2.0, a validated set of 1468 high-quality colour images. All were 512 × 512 pixels, 96 dpi, subtending 10° visual angle when viewed from a distance of 70 cm. Stimuli were presented using Psychopy V1.85.1.^59^ Each image was only used once as either a standard, rare oddball, or foil. To avoid repetition effects, a new selection of images were used for standards, oddballs and foils at 1 year retest. An example of the images used are provided in Fig. 1.

##### Standards

416 standard images [mean image intensity of 0.82 (SD = 0.28)], were presented once in a randomized order that differed across participants.

##### Oddballs

Eight oddball images [mean image intensity of 0.82 (SD 0.25)] were pre-selected. An equal number of natural and manmade objects were chosen to ensure no systematic semantic categorical distinction between standards and oddballs.

##### Foils and lures

For the pre-fastball encoding two alternative forced choice (2AFC) task, eight pictures [mean image intensity = 0.77 (SD 0.29)] were pre-selected as foils (previously unseen distractor images) and remained consistent across participants. For the post-Fastball 2AFC task, 16 semantically identical and perceptually similar images [mean image intensity of 0.80 (SD 0.29)] were used as lures to the eight oddball stimuli and eight pre-selected standard stimuli.

#### DMS-48 stimuli

**Figure 2:**
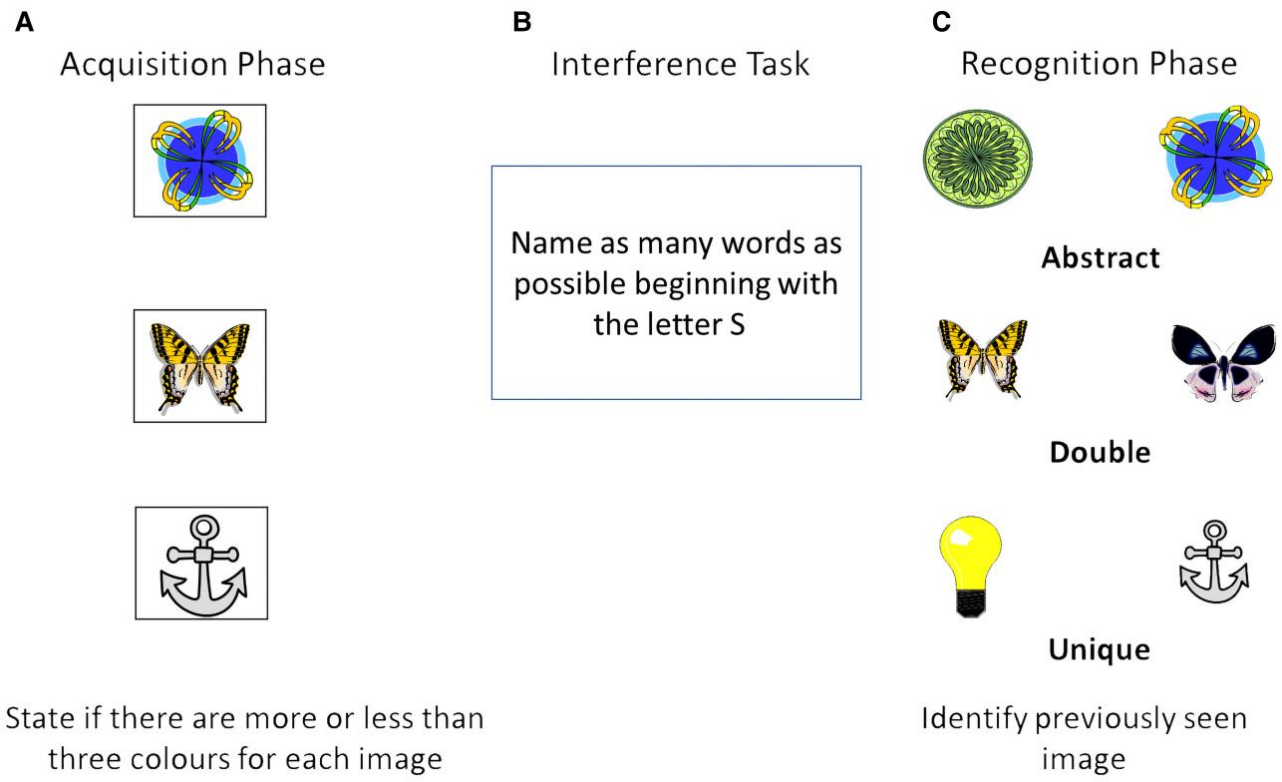
**Delayed matching to sample task− 48 (DMS-48).** (**A**) Participants were presented with 48 clip-art style images which they viewed sequentially and stated out loud whether each had more or <3 colours. (**B**) Participants then completed an interference task. (**C**) The targets were split into 3 groups of 16: (i) unique images- targets and foils are different, (ii) paired pictures- targets and lures belong to the same semantic category, (iii) abstract items- targets and distractors are abstract patterns.

The Delayed Matching to Sample task 48 (DMS-48) is a neuropsychological measure of visual recognition memory.^60^ It is composed of three sets of 48 coloured clipart style images (targets) which the participant encodes sequentially (acquisition phase) and three sets of 48 pairs of images (Set 1, 2 and 3), consisting of one previously viewed target image paired with a previously unseen image (recognition phase). For this study only Set 1 was used for the recognition phase to mirror the immediate recall used for the Fastball 2AFC task. The 48 target images are split into three groups of 16: 1) abstract items- targets and distractors are patterns that are difficult to verbalize; 2) paired pictures- targets and lures belong to the same semantic category and are alike in shape and colour; 3) unique images- targets and foils differ perceptually and semantically (see Fig. 2). For the year one follow-up we adapted the procedure and used the Set 2 distractors/foils/lures as the new targets and the distractors/foils/lures from Set 3 to avoid repetition.

#### Psychomotor vigilance task

The psychomotor vigilance task (PVT) is a commonly used measure of sustained attention that requires participants to respond to an unpredictable stimulus onset. PVT design followed that of Manuel *et al.*^61^ and was presented using Psychopy v1.85.1.^59^ The stimulus was an onscreen stopwatch in the form of numbers to one decimal place that began at zero with every stimulus onset. The numbers counted upwards in seconds and stopped as soon as the participant pressed the spacebar. The numbers were presented in Verdana font and subtended 7° of visual angle when viewed at a distance of 70 cm. The inter-stimulus period varied randomly between 2 and 10 s. Thirty stimuli were presented. Median response times were calculated per participant; any response faster than 100 ms was discarded and slower than 500 ms was classed as a lapse.

### Procedure

Participants completed the Fastball task followed by a neuropsychological battery to assess general cognition (ACE-III,^62^), vision (Freiberg Visual Acuity,^63^), attention (PVT) and recognition memory (DMS-48). The order of tasks was counterbalanced across participants. The same procedure was completed one year after the baseline session, but the stimuli were changed for the Fastball task and DMS-48, and a different version of the ACE-III was used to avoid learning effects.

#### Fastball

Participants were presented with eight pre-selected oddball images individually for 3 s, and were asked to name the image out loud. The image was then shown next to a foil and the participant had to indicate which image they had just seen using the left and right arrow keys. The position of the previously seen item was pseudo-randomized to ensure equal distribution between the left and right side of the screen. An incorrect response resulted in the participant receiving onscreen feedback asking them to try again. Participants were not able to move on to the next image until they had responded correctly. The purpose of object naming and making a discriminatory choice was to strengthen encoding of the image as the depth of processing has been shown to be vital for encoding.^64^

Images were displayed on-screen in sequences of five images, the first four were selected from the standard category and every fifth image from the oddball category. Images were presented for 166 ms with an inter-stimulus interval of the same duration. This method generates two distinct steady state responses; the visual presentation frequency at 3 Hz and the oddball frequency at 0.6 Hz. All standard stimuli were randomly selected from the standard image pool and shown only once. Oddball images were presented 13 times each in a pseudo-randomized order to avoid consecutive presentations. Overall, 520 images were presented in one trial, which lasted 173 s.

To avoid lapses in attention, which may occur in a purely passive task, participants were asked to maintain their gaze on a black fixation cross in the centre of the screen, and press a key in response to it turning red. This happened randomly on 10% of sequences and lasted for the duration of the sequence (1.66 s). All participants were able to understand the task instructions and respond using the keyboard. Speed and accuracy were recorded for each participant.

Immediately after the Fastball task participants completed a 2AFC task. 8 oddball images and 8 pre-selected standard images previously viewed during Fastball were paired with 16 semantically identical and perceptually similar lures. The participant was required to select which image they had seen during the experiment using the left and right arrow keys. Images stayed on-screen until a response was made, this was then immediately followed by another pair of images. In total the Fastball task took ∼5 min to complete.

#### DMS-48

Participants viewed 48 coloured clip-art images sequentially and stated out loud whether each image had more or <3 colours. Participants were kept to a 7-min time limit to ensure they had enough time to perceptually process the pictures. Participants then completed a 2-min lexical fluency interference task in which they were asked to say as many words as possible beginning with the letter ‘S’. Only Set 1 was used for the recognition phase. Targets and distractors were presented side by side and were equally distributed on the left and right. The targets were presented in the exact opposite order of presentation during encoding. The participant viewed each pair of images and selected either picture A or B to indicate which one they thought they had seen previously.

#### PVT

Participants were instructed to press a spacebar in response to numbers appearing on the screen. If participants responded in the absence of the numbers, they would receive on-screen feedback stating ‘false start’.

Tasks were well tolerated, and all participants were able to complete all the tasks. One patient's PVT data lost due to a technical error.

### EEG recording

Participants’ EEG was sampled at 500 Hz using an 8-channel Enobio gel sensor system (Neuroelectrics, Barcelona). An ear clip with two electrodes (common mode sense and driven right leg) served as a dual reference. Electrodes were placed at the following standard 10–10 locations; O1, P7, Pz, Cz, F3, F4, P8 and O2. The Neuroelectrics Instrument Controller (NIC2) software application was used to record data. Data were sent between Psychopy and NIC2 via LabStreamingLayer.^65^ Impedances were below 15 kΩ. Recordings were analysed offline using Brain Electrical Source Analysis software v5.3 (BESA GmbH) and custom built MATLAB code (Mathworks Inc.).

### EEG analysis

Data were analysed using the same procedure described in.^39,40^ Data were re-referenced offline to a common average reference, downsampled to 120 Hz. To avoid aliasing artefacts a 40 Hz 24 db zero-phase lowpass filter was applied. The steady-state response was calculated according to the procedures described in Stothart *et al*.^39^ Epochs from 0 to 173 s around trial onset were defined for each condition. This epoch length represents an integer number of cycles (104) of the oddball stimulus (0.6 Hz) ensuring that a frequency bin corresponding to the exact oddball frequency and its harmonics, including the standard frequency (3 Hz), was created. The frequency resolution was 0.0058 Hz. Epochs were polynomially de-trended to remove DC and slow-wave artefacts.

As we used single epochs of a long duration, visual inspection revealed occasional instances of gross artefacts, e.g. large physical movement artefacts. Any artefact ±250 uV was removed from the data and replaced with zeros. To avoid discontinuities in the remaining data, data on either side of any removed section was tapered to zero using half a Hanning window over 670 points of data. Across participants, the mean percentage of data removed by this procedure was 2.2% (SD = 8.4%).

For each subject and each electrode, amplitude was computed on these windows using the Fourier transform. SNR was then calculated by dividing the amplitude in each frequency bin by the mean amplitude of surrounding bins within a ± 0.10 Hz range^35,66,67^ excluding the immediately adjacent bins (first neighbouring bin on each side). Excluding the immediately adjacent bins from this correction meant that the amplitude correction was less likely to include any spread of the signal to proximal frequency bins.

Previous research has shown a robust SSVEP response to the oddball frequency and many of its harmonics^68^ with oddball detection more reliably and accurately measured when including the harmonics of the oddball response e.g.^67^ Consequently, the SNR was calculated for two values: the standard frequency **F** (3 Hz) and the mean of the oddball frequency and significant harmonics ***f +*** . To identify which harmonics to include in the calculation of ***f+***, group *Z* scores were calculated for each harmonic (based on the global average of all electrodes) relative to the neighbouring frequency bins within a ± 0.10 Hz range. This identified the highest significant harmonic (*Z* > 1.96) at 6.6 Hz for older adults, therefore for further analyses ***f +*** was calculated at each electrodes as the mean SNR of 0.6, 1.2, 1.8, 2.4, 3.6, 4.2, 4.8, 5.4 and 6.6 Hz.

### Statistical analysis

#### Sample size and power calculations

Comparisons between HOA and AD patients showed large effect sizes (Cohen's *D* = 1.35) To replicate this effect at *α* of 0.01 and a *β* of 0.95 required a sample size of *n* = 13. However, the effects of an earlier stage of MCI on Fastball recognition oddball responses were unknown and there were no previous studies on which to base between group effect size estimates, consequently sample sizes were tripled to ensure adequate power for subgroup analyses.

#### Between group comparisons

To examine the effect of group (HOA versus naMCI versus aMCI) on oddball responses (***f+***) while controlling for basic steady state magnitude (**F**), two ANCOVA models were used. The first included all eight electrodes in a 3 (group) × 8 (electrode) design, and the second used the maximal electrode for each participant only in a 1-way (group) design. The purpose of the second, simpler model was to capture individual differences in the topography of the oddball response. To examine the effect of group on visual steady-state responses (**F**) a 3 (group) × 8 (electrode) design ANOVA was used. The speed and accuracy of the detection of fixation cross colour changes were compared across groups using a 1-way (group) ANOVA. Parametric assumptions of distribution and homogeneity of variance were met for all ANOVA models.

Behavioural responses to the Post-Fastball 2AFC, DMS-48 and PVT tasks were not suitable for parametric statistics; therefore, Kruskall-Wallis tests were conducted for each condition to examine the effect of group on performance. *Post hoc* analyses of significant group effects were conducted using Bonferroni corrected Mann-Whitney U tests.

#### Linear regressions

The core outputs of the Fastball test (***f+***, Post-Fastball 2AFC recognition accuracy and RT) were combined in a linear model to predict neuropsychological performance amongst MCI patients. 95% confidence intervals and significance values were derived from 1000 bootstrap samples.

#### Test-retest reliability

To determine the consistency of Fastball responses in HOA across the two timepoints, we calculated the intraclass correlation coefficient (ICC) between participants’ scalp-average ***f +*** in session 1 and session 2 using a two-way mixed model; ICC(A, *k*) (absolute agreement) and ICC(C, *k*) (consistency) are reported. An *F*-test indicated bias within our model that did not reach statistical significance (*F* (1.46) = 3.59, *P* = 0.061) but was nonetheless present; therefore, a two-way model was deemed appropriate (Liljequist *et al*.). Consistent with previous FPVS test-retest reliability research^69^ we employed the following criterion for interpreting ICC values: coefficients below 0.40 are poor; 0.41 and 0.59 are moderate, 0.60 and 0.74 are good and coefficients above 0.75 are excellent. For transparency, both the single and multiple score ICC values are reported, as we propose FPVS responses could be used clinically, through a single measurement, or the mean of multiple measurements.^70^ As a comparator ICCs were also calculated for the ACE-iii memory subscale score.

## Results

### Fastball neural recognition performance

**Figure 3:**
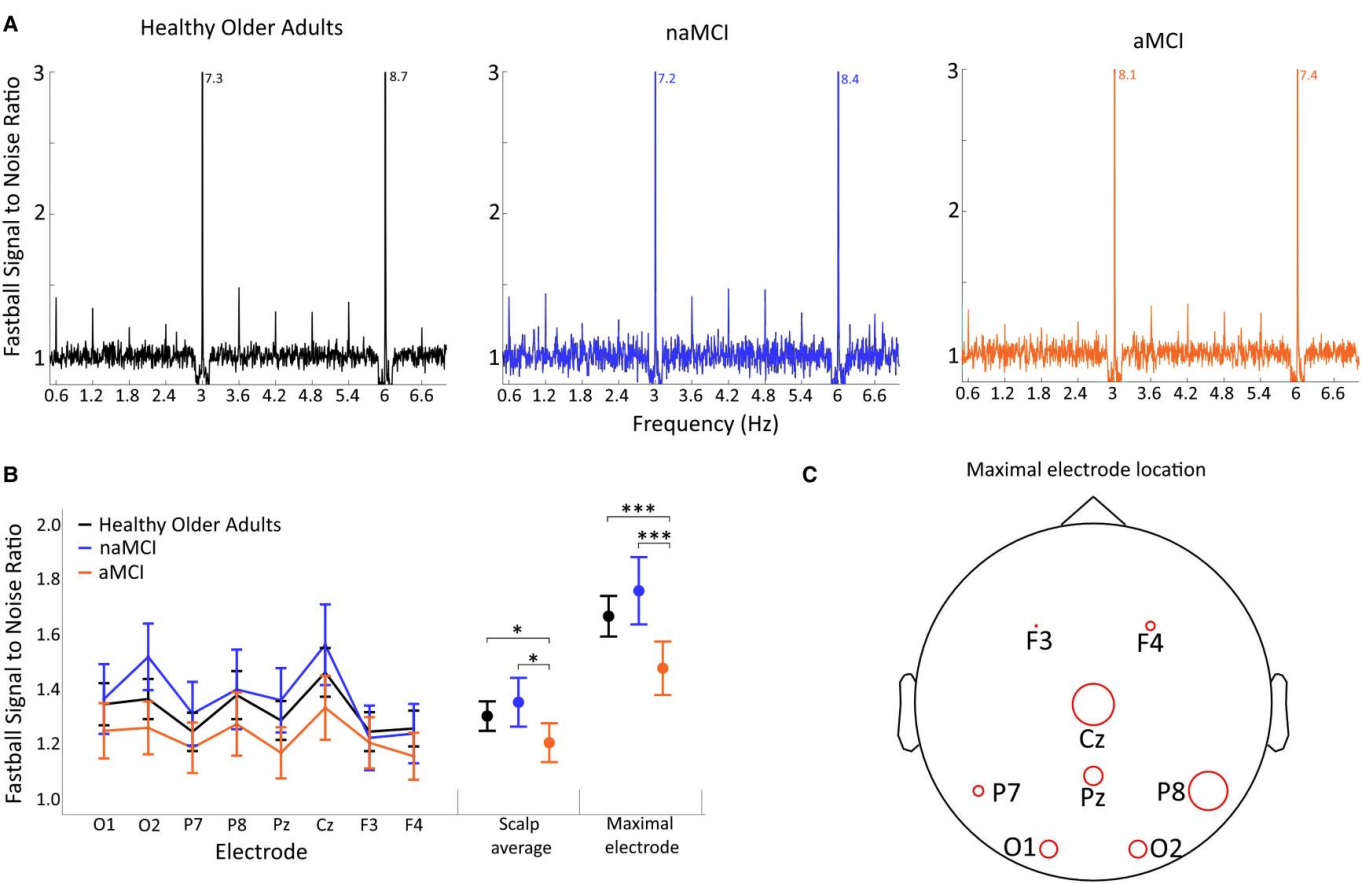
**Neural recognition responses.** (**A**) Spectral plots represent the Fastball SNR for the three groups (HOA *n* = 54, aMCI *n* = 33, naMCI *n* = 20), averaged across all electrodes and participants. Values for **F** are provided when larger than y axes maxima. (**B**) Fastball ***f +*** responses (estimated marginal means adjusted for F), averaged across participants for the three groups at individual electrodes, scalp average and maximal electrode. Error bars indicate 95% confidence intervals. ***P < 0.001, * *P* < 0.05 (significance values are taken from the 3 (group) × 8 (electrode) ANCOVA to determine the effect of group on oddball responses (f+) controlling for basic steady state magnitude (**F**) presented in full in Table 2). (**C**) Scalp plot showing single electrodes, at which largest ***f +*** responses occurred. Circle size corresponds to the proportion of participants, across all groups for whom the electrode showed the largest ***f +*** response.

aMCI patients showed reduced neural recognition memory responses compared with naMCI patients and HOA, see Fig. 3.

#### Main effects of group

Fastball responses differed significantly across groups with aMCI patients showing significantly reduced responses compared with naMCI and HOA, see Table 2. Figure 3C shows the location of the maximal electrode across groups, with Cz and P8 being the most common. There were no clear differences in maximal electrode distributions between groups, this data is presented in the Supplementary Information.

**Table 2:** 3 (group) × 8 (electrode) ANCOVA to determine the effect of group on oddball responses (***f+***) controlling for basic steady state magnitude (**F**), and *post hoc* pairwise comparisons of group

Main effects					
		df	*F*	*P*	Cohen's *D*
**All electrodes**	Group (HOA, naMCI, aMCI)	2103	3.67	0.029	0.44
	Electrode (O1, P7, Pz, Cz, F3, F4, P8, O2)	7721	1.07	0.384	0.02
	Group × Electrode	14 721	0.94	0.51	0.00
**Maximal electrode**	Group (HOA, naMCI, aMCI)	2103	6.91	0.002	0.66

*Post hoc* analyses of group effects												
	HOA versus naMCI				HOA versus aMCI				naMCI versus aMCI			
		95% CI	*P*	Cohen's *D*		95% CI	*P*	Cohen's *D*		95% CI	*P*	Cohen's *D*
**All electrodes**	−0.05	−0.02, 0.05	0.34	0.24	0.09	0.01, 0.18	0.038	0.46	0.14	0.03, 0.25	0.013	0.71
**Maximal electrode**	−0.09	−0.23, 0.048	0.20	0.34	0.17	0.05, 0.29	0.005	0.64	0.27	0.11, 0.42	0.001	0.98

### Post-fastball 2AFC behavioural recognition performance

#### Recognition of standard images

**Figure 4:**
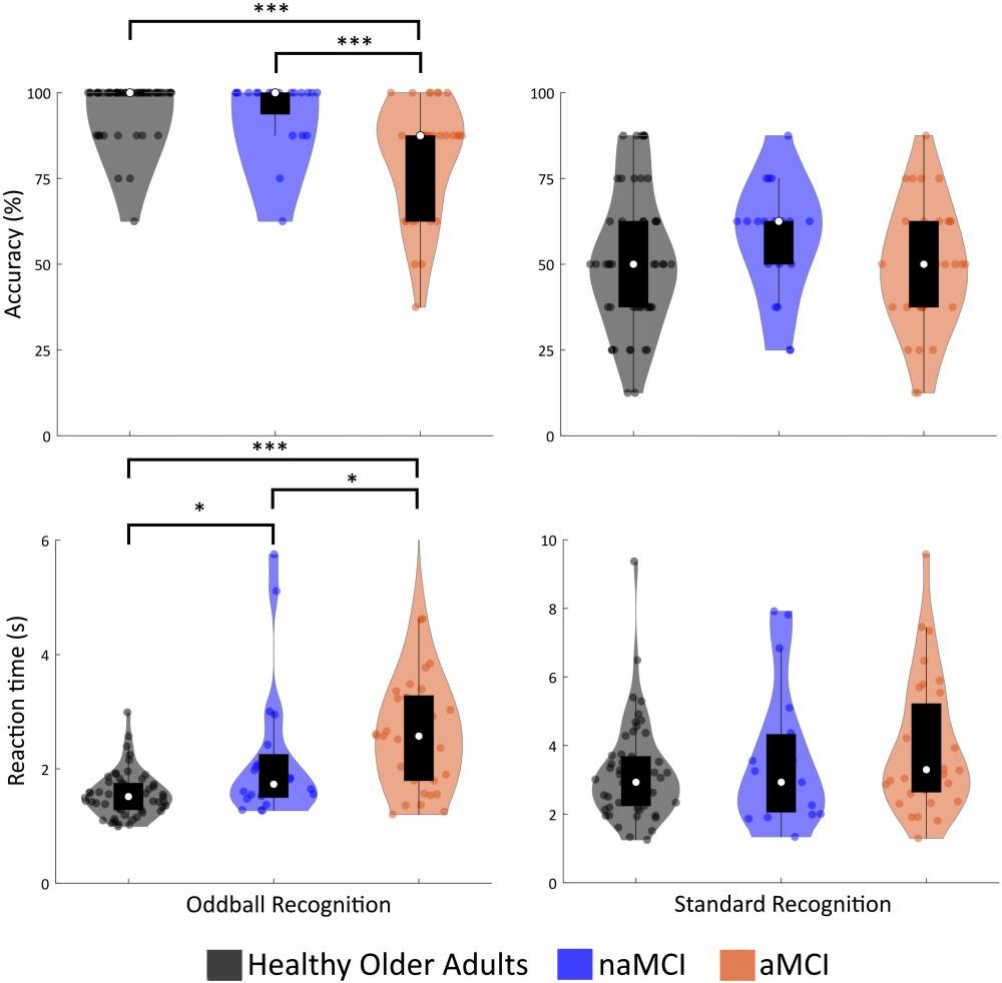
**Behavioural recognition responses violin plots illustrating behavioural recognition accuracy (%) and reaction time (S) to the post-Fastball 2AFC task, (HOA *n* = 54, aMCI *n* = 33, naMCI *n* = 20).** Individual data points reflect the correct recognition accuracy or reaction time of either an oddball or a standard stimulus compared with a lure. Tukey boxplots reflect the median and inter-quartile ranges, width of the violinplots reflects kernel density estimated using Matlab's ksdensity function. ***P < 0.001, *P < 0.05 (significance values are taken from the statistical tests presented in full in Post-Fastball 2AFC behavioural recognition performance—Recognition of oddball images).

Participants performed at chance with no significant group differences in accuracy [*H* (2, *n* = 107) = 1.82, *P* = 0.403] or reaction time [*H* (2, *n* = 107) = 4.41, *P* = 0.110], see Fig. 4, although aMCI patients appear to show greater variability in their reaction times.

#### Recognition of oddball images

There was a significant effect of group on accuracy [*H* (2, *n* = 107) = 32.89, *P* < 0.001]. aMCI patients were less accurate than HOA (*U* = 344, *Z* = −5.47, *P* < 0.001), and naMCI patients (*U* = 147, *Z* = −3.56, *P* < 0.001). naMCI patients and HOA did not significantly differ in accuracy (*U* = 518, *Z* = −0.53, *P* = 0.597).

There was a significant effect of group on reaction time [*H* (2, *n* = 107) = 29.63, *P* < 0.001]. aMCI patients were slower than HOA (*U* = 289, *Z* = −5.33, *P* < 0.001), and naMCI patients (*U* = 212, *Z* = −2.17, *P* = 0.03). naMCI patients were also slower than HOA (*U* = 347, *Z* = −2.43, *P* = 0.015).

### Visuo-attentional performance

#### Visual steady state response

There were no differences between groups in visuo-attentional engagement (**F**) with the task as indexed by the magnitude of the 3 Hz steady-state response to image presentation [**F**(2104) = 0.65, *P* = 0.525]. There was a significant effect of electrode [**F**(7728) = 22.87, *P* < 0.001)] with occipital electrodes showing the highest steady-state magnitudes and no significant group × electrode interaction [**F**(14 728) = 1.37, *P* = 0.162)].

#### Fixation cross colour change detection

There was no significant difference in accuracy between Older Adults (mean accuracy 93% ±16), naMCI patients (mean accuracy 88% ± 22) and aMCI patients (mean accuracy 92% ± 19), [**F**(2103) = 0.60, *P* = 0.553]. aMCI (mean RT 573 ms ± 201 ms) and naMCI patients (mean RT 614 ms ± 198 ms) were slower to responded to target crosses than HOA (mean RT 499 ms ± 88 ms), but did not differ significantly between themselves [**F**(2103) = 4.98, *P* = 0.009].

### Neuropsychological performance

#### DMS-48

There was a significant effect of group on accuracy [*H* (2, *n* = 107) = 45.35, *P* < 0.001]. aMCI patients were less accurate than HOA (*U* = 176, *Z* = −6.34, *P* < 0.001), and naMCI patients (*U* = 132, *Z* = −3.64, *P* < 0.001). naMCI patients were less accurate than HOA (*U* = 301, *Z* = −2.99, *P* = 0.003).

#### PVT

##### Lapses

There was a significant effect of group on accuracy [*H* (2, *n* = 106) = 25.3, *P* < 0.001]. aMCI patients (mean = 5.4 ± 5.1) showed significantly more lapses than HOA (mean = 1.4 ± 2.0) (*U* = 334, *Z* = −5.05, *P* < 0.001). naMCI patients also showed significantly more lapses (mean = 3.8 ± 5.0) than HOA (*U* = 371, *Z* = −2.22, *P* = 0.026). Amnestic and naMCI patients did not differ significantly in the number of lapses, (*U* = 243, *Z* = −1.61, *P* ≤ 0.109).

##### Reaction time

There was a significant effect of group on reaction time [*H* (2, *n* = 106) = 9.85, *P* = 0.007)]. aMCI patients showed significantly slower reaction times (mean = 413 ms, ±141) than HOA (mean = 354 ms, ±31) (*U* = 556, *Z* = −3.03, *P* = 0.002). naMCI patients did not show significantly slower reaction times (mean = 375, ±58) than HOA (*U* = 418, *Z* = −1.58, *P* = 0.115) or aMCI patients (*U* = 268, *Z* = −1.14, *P* = 0.255).

### Fastball and neuropsychological performance

Fastball neural responses (***f+***) significantly and selectively predicted memory performance on the ACE-iii. Behavioural accuracy on the post-Fastball 2AFC also significantly predicted memory performance, but less selectively as it also significantly predicted attentional performance, see Table 3. Behavioural accuracy was a significant predictor of DMS-48 performance, whereas Fastball neural responses (***f+***) only predicted performance on the abstract subscale, and not beyond the multiple comparison threshold.

**Table 3:** Linear regression statistics for fastball metrics and overall model performance in predicting MCI patients’ ACE-iii scores and DMS-48 scores.

	Addenbrookes Cognitive Exam iii																	
	Total			Memory			Attention			Verbal Fluency			Language			Visuospatial		
	*β* (Std. *β*)	95% CI	*P*	*β* (Std. *β*)	95% CI	*P*	*β* (Std. *β*)	95% CI	*P*	*β* (Std. *β*)	95% CI	*P*	*β* (Std. *β*))	95% CI	*P*	*β* (Std. *β*)	95% CI	*P*
***f+***	10.75 (0.34)	3.47, 19.57	0.009	**6.46 (0.35)**	**2.71, 10.65**	**0.004**	0.31 (0.04)	−2.01, 2.38	0.791	2.50 (0.31)	0.42, 5.10	0.033	1.23 (0.18)	−0.70, 3.90	0.278	0.43 (0.08)	−0.87, 1.71	0.479
**Accuracy**	**2.97 (0.40)**	**1.14, 4.34**	**0.002**	**2.00 (0.47)**	**1.07, 2.82**	**<0.001**	**0.90 (0.53)**	**0.36, 1.42**	**0.002**	0.19 (0.12)	−0.29, 0.63	0.321	−0.35 (−0.22)	−0.71, −0.06	0.041	0.08 (0.07)	−0.38, 0.44	0.677
**Speed**	−1.87 (−0.25)	−4.68, −0.45	0.018	−1.18 (−0.28)	−2.15, −0.11	0.017	−0.12 (−0.07)	−0.59, 0.16	0.451	−0.30 (−0.16)	−1.19, 0.19	0.230	−0.11 (−0.07)	−0.56, 0.34	0.527	−0.18 (−0.14)	−1.21, 0.89	0.458
**Model (df** = **3.49)**	***r^2^***	**F**	***P***	***r^2^***	**F**	***P***	***r^2^***	**F**	***P***	***r^2^***	**F**	***P***	***r^2^***	**F**	***P***	***r^2^***	**F**	***P***
	**0.44**	**12.77**	**<0.001**	**0.54**	**19.18**	**<0.001**	**0.31**	**7.29**	**<0.001**	0.16	3.05	0.037	0.06	1.15	0.340	0.04	0.65	0.586

	Delayed Match to Sample 48												Psychomotor Vigilance Task					
	Total			Unique			Paired			Abstract			Lapses			Reaction time		
	*β* (Std. *β*)	95% CI	*P*	*β* (Std. *β*)	95% CI	*P*	*β* (Std. *β*)	95% CI	*P*	*β* (Std. *β*)	95% CI	*P*	*β* (Std. *β*)	95% CI	*P*	*β* (Std. *β*)	95% CI	*P*
***f+***	3.22 (0.15)	−1.46, 8.85	0.206	0.45 (0.06)	−1.03, 2.34	0.58	−0.20 (−0.02)	−1.99, 2.08	0.872	2.97 (0.31)	0.73, 5.73	0.021	−5.13 (−0.23)	−11.52, −0.71	0.076	−0.03 (−0.15)	−0.08, 0.016	0.172
**Accuracy**	**2.70 (0.52)**	**1.04, 4.06**	**0.002**	**0.73 (0.43)**	**0.26, 1.12**	**0.001**	**1.21 (0.60)**	**0.34, 1.85**	**0.005**	0.76 (0.33)	0.03, 1.49	0.034	−0.87 (−0.24)	−1.85, 0.38	0.115	−0.01 (−0.26)	−0.03, 0.01	0.121
**Speed**	−0.38 (−0.08)	−1.67, 1.19	0.517	−0.36 (−0.22)	−0.74, 0.14	0.085	0.085 (0.04)	−0.30, 0.78	0.681	−0.11 (−0.05)	−0.74, 0.63	0.690	0.77 (0.21)	−0.06, 2.48	0.220	0.01 (0.19)	0.00, 0.03	0.111
**Model (df** = **3.49)**	***r^2^***	**F**	***P***	***r^2^***	**F**	***P***	***r^2^***	**F**	***P***	***r^2^***	**F**	***P***	***r^2^***	**F**	***P***	***r^2^***	**F**	***P***
	0.34	8.50	<0.001	0.29	6.55	0.001	0.35	8.73	<0.001	0.24	5.24	0.003	0.18	3.70	0.018	0.16	3.13	0.034

Accuracy and Speed are derived from the post-task 2AFC performance. 95% confidence intervals and significance values are derived from 1000 bootstrap samples. Beta values (*β*) are presented with standardized beta values (*Std. β*). Values in bold indicate statistically significant results after Bonferroni correction for multiple comparisons (ACE-ii, 0.05/6, *P* = 0.008; DMS-48, 0.05/4, *P* = 0.0125; PVT, 0.05/2, *P* = 0.025)

### Year one follow-up and retest

52 HOA and 42 patients repeated the study procedure after 1 year to establish the stability of Fastball responses in HOA, and the sensitivity of Fastball to cognitive decline in patients. At year one follow-up 36 patients remained as MCI, four aMCI and two naMCI patients had since converted to AD and two aMCI patients had converted to vascular dementia. Table 4 indicates marginal or no change in ACE-iii scores at year one follow-up. Patients who had converted to either AD or Vascular dementia showed a trend for impaired neural and behavioural Fastball performance at baseline that increased at follow-up, with little or no change in ACE-iii scores. Given the low number of converters to dementia caution must be taken in overinterpreting these trends and it was not meaningful to statistically compare converters versus non-converters.

**Table 4:** Fastball and ACE-iii scores at baseline and year one follow-up for MCI patients, grouped by year one clinical status

	Fastball						ACE-iii			
	*f+*		2AFC Accuracy		2AFC Speed		Total score		Memory subscale	
	Baseline	Year one follow-up	Baseline	Year one follow-up	Baseline	Year one follow-up	Baseline	Year one follow-up	Baseline	Year one follow-up
**MCI-stable (*n*** = **36)**	1.31 (0.23)	1.25 (0.18)	7.03 (1.34)	6.86 (1.22)	2.41 (1.15)	2.74 (1.80)	81.97 (10.07)	79.89 (11.39)	18.14 (5.45)	16.81 (5.36)
**MCI-AD converter (*n*** = **6)**	1.21 (0.23)	1.13 (0.11)	6.33 (1.21)	7.17 (0.98)	2.77 (0.97)	4.06 (2.38)	77.67 (7.66)	77.33 (9.73)	16.67 (6.22)	16.00 (6.51)
**MCI-VaD converter (*n*** = **2)**	1.23 (0.09)	1.10 (0.01)	6.50 (2.12)	6.00 (2.83)	1.80 (0.34)	1.54 (0.37)	80.00 (9.90)	83.50 (7.78)	15.00 (7.07)	16.50 (7.78)

#### Test-retest reliability in HOA

Test-retest reliability using single measure ICCs was moderate for Fastball responses, and good if using average measure ICCs. In both examples test-retest reliability was higher than the ACE-iii memory subscale scores, see Table 5.

**Table 5:** Two-way model of intraclass correlations to determine the consistency of fastball responses (***f+***) between baseline and year one follow-up.

	Maximal electrode *f+*		ACE-iii memory subscale score	
	Consistency	Absolute agreement	Consistency	Absolute agreement
**single**	0.58 [0.37, 0.74]	0.56 [0.33, 0.72]	0.38 [0.12, 0.59]	0.38 [0.12, 0.59]
**average**	0.74 [0.54, 0.85]	0.72 [0.50, 0.84]	0.55 [0.22, 0.74]	0.55 [0.22, 0.74]

Single measures correspond to the reliability of oddball responses recorded during individual Fastball sessions, whilst average measures correspond to the reliability of an average of *k* sessions. Numbers in square brackets indicate 95% confidence intervals.

## Discussion

Fastball is a passive, objective measure of recognition memory that is sensitive and specific to amnestic impairment in MCI. aMCI patients showed reduced implicit neural (***f+***) and explicit behavioural (Post-Fastball 2AFC accuracy) recognition memory performance compared with naMCI and HOA. There were no group differences in basic visual processing (**F**) or fixation cross colour change detection accuracy. Patients were slower than controls at responding to the fixation cross colour change, but aMCI and naMCI patients did not differ. Neuropsychological assessment (DMS-48) confirmed an explicit recognition memory deficit specific to aMCI, while sustained attention (PVT) was impaired in MCI patients as a group but not specifically in aMCI. Regression analyses showed Fastball neural responses to be specifically predictive of neuropsychological measures of memory function and not other cognitive functions. Fastball behavioural responses predicted both memory and attentional function. Intra-class correlations of Fastball responses between baseline and year one follow-up testing in HOA showed moderate to good reliability that outperformed the ACE-iii memory subscale.

We propose that Fastball is an implicit marker of familiarity, a cognitive function repeatedly linked to the perirhinal cortex.^71,72^ Given the perirhinal cortex is one of earliest affected areas of the brain in AD, an accurate method for measuring familiarity processing could provide a valuable early cognitive marker of AD. In the current study aMCI patients who subsequently converted to AD showed lower Fastball responses at baseline. Numbers of converters at year one follow-up were small so this is no more than a promising trend that will be further investigated with annual follow-ups, nevertheless it is in line with Bastin *et al.*'s^56^ recent longitudinal study of MCI-AD converters that showed aMCI patients who went on to convert to AD had clear deficits in familiarity performance at baseline.

An open question following our demonstration of Fastball reductions in AD^40^ was whether deficits in sustained attention, rather than recognition memory, contributed to patients’ reduced responses. The data presented in the current study help to reject this possibility. MCI patients as a group showed impaired sustained attention (PVT) performance relative to HOA, but aMCI and naMCI were not significantly different. This pattern was also observed in the reaction times to the fixation cross colour change. Regression analyses also showed no strong relationship between ***f +*** and either the attention subscale of the ACE-iii or PVT performance. Note however, that there was a significant linear relationship between ***f +*** and the memory subscale of the ACE-iii, i.e. while behavioural recognition accuracy may be confounded by attention it is possible that the implicit neural measure (***f+***) was not. Attentional deficits may therefore be a useful general marker of cognitive impairment, but do not appear to be useful for identifying the amnestic deficits that are likely to be early markers of AD. MCI patients’ reaction times to the 2AFC Post-Fastball 2AFC behavioural recognition performance were more variable, especially when identifying previously seen standard images. This suggests a noisier, less efficient decision making process; however, given the low number of trials, we are cautious in over-interpreting this effect.

Fastball can detect amnestic deficits in MCI passively, and objectively and has good face validity as a memory assessment tool, predicting composite memory function (ACE-iii memory subscale) and specific recognition memory performance (DMS-48). Because the core elements of the task are passive, the technique has the potential to avoid confounds of educational, developmental, anxiety, linguistic and cultural biases that are known to significantly affect performance on traditional neuropsychological assessments.^26-29,31,73^ Further studies are required to empirically demonstrate these advantages. Fastball requires cheap, non-invasive scalable EEG technology, giving it significant advantages over other functional biomarker technologies such as functional MRI and PET. We do not propose that Fastball could, or even should, replace standard pen and paper cognitive testing. Rather we propose that an implicit neural measure has many potential practical advantages and with sufficient technological development could be delivered and interpreted as easily as an electrocardiogram, which is routinely delivered economically, at scale, with minimal training required. Fastball responses demonstrated moderate to good consistency and absolute agreement between baseline and year-one retests in HOA. Importantly, consistency and absolute agreement values were similar, indicating that Fastball measures are reliable when potential biases are considered. In practice, this suggests that responses remain stable in cognitively healthy individuals and can be expected to fall within a similar range for the same person across sessions.

Dementia blood biomarkers provide the possibility of truly scalable dementia screening and triaging in the near future. While progress has been impressively, fast blood biomarkers in isolation will not be a panacea for dementia diagnosis. Co-morbidities, sex, race, ethnicity, even circadian rhythms have been shown to affect blood biomarker accuracy,^74,75^ and it is most likely that the combination of measures of brain structure and function will be more powerful than either alone. Including cognitive function as a core feature of AD is still considered integral by a significant proportion of the dementia clinical research community.^15^

PET and CSF biomarkers were not available in the current study, which limited our ability to identify the patients at baseline most likely to develop AD, instead longitudinal clinical follow-up was used to retrospectively group patients at baseline. However, the follow-up period of one year was too short to meaningfully interpret the prognostic value of Fastball as too few patients had converted to dementia. These patients will continue to be followed up, and the prognostic value of a baseline Fastball assessment established. Future studies would benefit from pairing Fastball with established structural biomarkers in order to explore the relationship between brain structure and recognition memory function.

In summary, we present further validation of Fastball as a new method for objectively measuring visual recognition memory in MCI. Fastball is sensitive to amnestic impairment in MCI unconfounded by sustained attention ability. The prognostic value remains to be established, but its passive, objective properties give it significant potential as a diagnostic tool, and its use of cheap, portable technology brings significant practical advantages.

## Supplementary Material

fcaf279_Supplementary_Data

## Data Availability

The complete anonymized data and associated code is available on the on the Open Science Framework https://osf.io/nw37b/.
