# Correction to: A passive and objective measure of recognition memory in mild cognitive impairment using Fastball memory assessment

**DOI:** 10.1093/braincomms/fcag147

**Published:** 2026-04-24

This is a correction to: George Stothart, Sophie Alderman, Oliver Hermann, Sam Creavin, Elizabeth J Coulthard, A passive and objective measure of recognition memory in mild cognitive impairment using Fastball memory assessment, *Brain Communications*, Volume 7, Issue 5, 2025, fcaf279, https://doi.org/10.1093/braincomms/fcaf279

In the originally published version of this manuscript, the Competing Interests statement originally read, “The authors report no competing interests.” In the corrected article, the Competing Interests statement has been amended to read as follows: “GS is currently employed by The University of Bath and Cumulus Neuroscience Ltd. Cumulus Neuroscience Ltd had no involvement in this study. All other authors have no competing interests to declare.”

