# Supplementary material for: A passive and objective measure of recognition memory in mild cognitive impairment using Fastball memory assessment
Source: Brain Commun. 2025 Sep 1;7(5):fcaf279. doi: 10.1093/braincomms/fcaf279 (PMC12401560; doi:10.1093/braincomms/fcaf279)
Supplement: fcaf279_Supplementary_Data [file fcaf279_supplementary_data.docx]

# Supplementary Information

| Electrode | HOA | | aMCI | | naMCI | | Total | |
| --- | --- | --- | --- | --- | --- | --- | --- | --- |
|  | Count | Proportion | Count | Proportion | Count | Proportion | Count | Proportion |
| O1 | 6 | 0.11 | 5 | 0.15 | 1 | 0.05 | 12 | 0.11 |
| P7 | 3 | 0.06 | 4 | 0.12 | 0 | 0.00 | 7 | 0.07 |
| Pz | 7 | 0.13 | 3 | 0.09 | 3 | 0.15 | 13 | 0.12 |
| Cz | 14 | 0.26 | 9 | 0.27 | 6 | 0.30 | 29 | 0.27 |
| F3 | 1 | 0.02 | 0 | 0.00 | 0 | 0.00 | 1 | 0.01 |
| F4 | 5 | 0.09 | 1 | 0.03 | 0 | 0.00 | 6 | 0.06 |
| P8 | 14 | 0.26 | 8 | 0.24 | 5 | 0.25 | 27 | 0.25 |
| O2 | 4 | 0.07 | 3 | 0.09 | 5 | 0.25 | 12 | 0.11 |
| Total | 54 |  | 33 |  | 20 |  | 107 |  |

Supplementary Table 1: Maximal electrode locations of ***f+*** by group.
